# Lung Manifestation of Dengue Fever: A Retrospective Study

**DOI:** 10.7759/cureus.60655

**Published:** 2024-05-20

**Authors:** Lakshmi B Keshav, Adithi K, Karan Malhotra, Shraddha Shetty

**Affiliations:** 1 Medicine, Nottingham University Hospitals NHS Trust, Nottingham, GBR; 2 General Medicine, K. S. Hegde Medical Academy, Mangalore, Mangalore, IND; 3 Anesthesiology, Maharishi Markandeshwar Institute of Medical Sciences and Research, Ambala, IND; 4 Community Medicine, K. S. Hegde Medical Academy, Mangalore, Mangalore, IND

**Keywords:** mortality, acute respiratory distress syndrome, pleural effusion, dengue hemorrhagic fever, dengue fever

## Abstract

Introduction

Dengue fever, caused by the dengue virus transmitted by Aedes aegypti mosquitoes, is a significant public health concern globally. Its resurgence in recent years, particularly in low- and middle-income countries, has led to increased morbidity and mortality rates. Atypical manifestations, involving the cardiac, liver, gut, renal, blood, bone, nervous, and respiratory systems, in dengue, can complicate both diagnosis and management. This study aimed to investigate the incidence of lung manifestations in dengue-infected individuals and their correlation with patient outcomes.

Background

The prevalence of dengue fever has risen dramatically over the past two decades, with Asia bearing the brunt of the burden, particularly India. The pathophysiology of lung complications in dengue remains unclear but is thought to be related to capillary leak syndrome and thrombocytopenia. Studies suggest that respiratory symptoms may be associated with severe cases and increased mortality rates. Despite limited research in India, understanding lung manifestations in dengue is crucial for improving diagnostic accuracy and patient care.

Methods

A retrospective study was conducted at K.S. Hegde Hospital, a tertiary care facility located in Mangalore, India, involving patients aged 18 years and above diagnosed with dengue fever between January and December 2019. Data gathered comprised patient demographics, clinical symptoms, laboratory findings, imaging results including radiographs, computed tomography (CT) scans of the chest (if accessible), ultrasound examinations of the chest and abdomen, and 2D echocardiograms, as well as patient outcomes. Diagnosis of lung manifestation was established through clinical assessment, chest X-ray interpretation, and ultrasound of the chest. Statistical analysis was conducted using SPSS Statistics (version 20), with a significance set at p<0.05.

Results

Out of 255 dengue cases, 10.19% (n=26) exhibited pulmonary manifestations, with pleural effusion being the most common. Older age (>50 years) and comorbidities were associated with a higher incidence of lung involvement. Respiratory symptoms, such as breathlessness, were more prevalent in patients with pulmonary complications. Laboratory parameters indicated distinct profiles in patients with lung manifestations, including elevated total count, urea, bilirubin, and liver enzymes, and reduced platelet counts. Mortality rates were higher in patients with lung involvement, older age, and comorbidities.

Discussion

The study findings highlight the importance of recognizing respiratory symptoms in dengue fever, particularly in older patients and those with underlying health conditions. The association between pulmonary involvement and adverse outcomes underscores the need for early detection and appropriate management strategies. Future research should focus on elucidating the pathophysiology of lung complications in dengue and developing targeted interventions to improve patient outcomes.

Conclusion

Lung manifestations in dengue fever represent a significant clinical challenge and are associated with increased morbidity and mortality. Early recognition of respiratory symptoms, along with prompt diagnostic evaluation and appropriate management, is essential for improving patient prognosis. Further studies are warranted to deepen our understanding of lung involvement in dengue and optimize therapeutic approaches to mitigate its impact on patient outcomes.

## Introduction

Dengue fever, a viral disease transmitted by Aedes Aegypti mosquitoes, represents the most common arboviral infection affecting humans. It is caused by any of the four distinct serotypes (dengue virus, DENV; i.e., DENV 1-4) [1]. Over the last two decades, the World Health Organization (WHO) has observed a staggering eight-fold increase in reported dengue cases, rising from 505,430 cases in 2000 to over 5.2 million in 2019 [2]. Between 2000 and 2015, there was a notable increase in reported deaths, with numbers climbing from 960 to 4,032, indicating a significant rise in mortality rates [3]. Asia accounts for 70% of the total cases, with India contributing 34% [4]. The resurgence of dengue on a global scale is attributable to unplanned urbanisation, rising migration trends, and inadequate awareness, resulting in significant public health, social, and economic challenges for many low- and middle-income countries [5]. Individuals afflicted with this disease may present with atypical manifestations, including pulmonary involvement, which can complicate the diagnostic process.

The distinguishing pathophysiological feature between dengue fever and dengue hemorrhagic fever (DHF) lies in increased capillary permeability, plasma leakage, and haemorrhage. These mechanisms are also implicated in the development of atypical manifestations of dengue, including respiratory symptoms [6]. Respiratory manifestations of dengue may manifest as pleural effusion (unilateral/bilateral), acute respiratory distress syndrome (ARDS), non-cardiogenic pulmonary oedema, pneumonitis, and pulmonary haemorrhage [7].

The precise pathophysiology underlying lung complications in dengue remains elusive, yet they appear to coincide with capillary leak syndrome and thrombocytopenia [8]. Severe dengue cases have revealed inflammatory changes and lung damage on autopsy, accompanied by alveolar oedema and haemorrhage. Viral antigens have been identified in lung tissue samples from both experimental infections and autopsy specimens [9].

Patients with dengue may present primarily with respiratory symptoms, posing diagnostic challenges for physicians and highlighting the importance of cautious diagnosis. Studies suggest that the majority of patients experiencing fatal infections show lung complications, potentially contributing to mortality rates [10,11].

Given the limited research in India on this topic, we conducted a study to determine the incidence of lung manifestations in dengue infections and their correlation with patient outcomes.

## Materials and methods

We conducted a retrospective,record-based observational study at K. S. Hegde Hospital, a tertiary care facility situated in the coastal city of Mangalore, India. The study included patients aged 18 years and above who were admitted to the hospital between January 2019 and December 2019, with confirmed clinical and laboratory evidence of dengue fever. Dengue fever was diagnosed based on the presence of acute febrile illness, along with two or more of the following symptoms: fever, headache, nausea, vomiting, retro-orbital pain, myalgia, arthralgia, rash, or haemorrhagic manifestations, in conjunction with either a positive non-structural protein 1 (NS1) antigen by enzyme-linked immunosorbent assay (ELISA) or anti-dengue immunoglobulin M (IgM) antibody during the study period. Patients with suspected clinical dengue without laboratory confirmation and those with incomplete medical records were excluded from the study.

Approval for the study was obtained from the institutional ethics committee of K. S. Hegde Medical Academy (INST.EC/EC/079/2020-21). Hospital records of all patients diagnosed with dengue fever were reviewed to collect baseline characteristics and clinical history, including age, sex, comorbidities, duration of illness, fever, myalgia, arthralgia, headache, retro-orbital pain, bleeding manifestations, cough, breathlessness, haemoptysis, shock, and mortality. Laboratory data included complete blood count, renal function tests, liver function tests, chest X-ray, CT chest (if available), ultrasound of the chest and abdomen, and 2D Echocardiogram conducted on admission. Any subsequent chest X-rays or ultrasound scans performed during hospitalization were also examined. Diagnosis of lung manifestation was based on clinical assessment, chest X-ray, and ultrasound findings.

Statistical analysis was conducted utilizing Statistical Package for the Social Sciences (SPSS, version 20; IBM SPSS Statistics for Windows, Armonk, NY). Qualitative data were represented in terms of frequency and percentage, while quantitative data were presented as mean and standard deviation. The statistical significance between the two outcome groups was assessed using Student's T-test. The association between lung manifestations and outcome was evaluated using the chi-square test. Additionally, laboratory parameters were compared with pulmonary complications using the Mann-Whitney U test. A significance level of p < 0.05 was deemed statistically significant.

## Results

Our study comprised a total of 255 patients with clinically and laboratory-confirmed dengue. The majority of the patients were male (n = 117, 69.4%), and most fell within the age group of under 50 years (n = 209, 82%). Pulmonary manifestations were observed in 26 patients (10.19%) diagnosed with dengue fever. Among the study population, the majority were admitted with classical dengue fever (n = 239, 93.7%), while 14 patients (5.49%) met all four criteria for the diagnosis of DHF.

Table 1 presents a comparison of baseline characteristics between patients with and without pulmonary manifestations. Patients aged over 50 years diagnosed with dengue (p = 0.02) and those with comorbidities (p < 0.01) showed a higher incidence of pulmonary manifestation.

**Table 1:** Baseline characteristics of patients with dengue *Data were analysed using the chi-square test to assess the association between demographic/comorbidity variables and pulmonary complications. p<0.05 was deemed statistically significant. The data have been represented as n, %.

Baseline characteristics	Patients without pulmonary involvement, n=229 (%)	Patients with pulmonary involvement, n=26 (%)	Total, n=255 (%)	*P value
Age				
≤ 50 years	192 (83.8)	17 (65.4)	209 (82)	0.02
>50 years	37 (16.2)	9 (34.6)	46 (18)	
Sex				
Female	71 (31.0)	7 (26.9)	78 (30.6)	0.66
Male	158 (69.0)	19 (73.1)	177 (69.4)	
Comorbidities				
No	194 (84.7)	14 (53.8)	208 (81.6)	<0.01
Yes	35 (15.3)	12 (46.2)	47 (18.4)	
Classification of dengue				
Classical dengue	215 (93.9)	24 (92.3)	239 (93.7)	>0.05
Dengue hemorrhagic fever	13 (5.7)	1 (3.8)	14 (5.5)	
Dengue shock syndrome	1 (0.4)	1 (3.9)	2 (0.8)	

Table 2 displays the relationship between various comorbidities and pulmonary involvement, assessed using the chi-square test. It reveals a significant association between pulmonary complications and conditions such as diabetes (p < 0.001), hypertension (p < 0.001), and chronic respiratory disease (p-value = 0.008). Hence, these comorbidities are notably linked to an increased likelihood of pulmonary involvement.

**Table 2:** Association between comorbidities and pulmonary involvement The data have been represented as n, %; p<0.05 is considered statistically significant.

Comorbidities	Patients without pulmonary involvement, n (%)	Patients with pulmonary involvement, n (%)	Total n (%)	Chi-square value	P value
Diabetes	17 (7.4%)	8 (30.8%)	25 (9.8%)	14.31	<0.001
Hypertension	17 (7.4%)	4 (15.4%)	21 (8.2%)	1.95	<0.001
Chronic Respiratory Disease	2 (0.9%)	2 (7.7%)	4 (1.6%)	7.03	0.008
Chronic Cardiac Disease	2 (0.9%)	1 (3.8%)	3 (1.2%)	1.77	0.183
Chronic Kidney Disease	2 (0.9%)	1 (3.8%)	3 (1.2%)	1.77	0.183

Table 3 outlines clinical presentations observed in the study population. Fever was the most common symptom, present in all patients with dengue. Most presentations were similar between patients with and without pulmonary complications, although a higher proportion of patients with pulmonary complications reported breathlessness (p < 0.01). Cough and haemoptysis were among other respiratory complaints noted in patients with dengue, with 14.8% (n = 34) of patients without pulmonary involvement reporting cough. It is worth noting that myalgia appears to be more prevalent among patients with pulmonary involvement compared to those without (p = 0.01).

**Table 3:** Clinical presentation of patients with dengue *Data were analysed using chi-square analysis to link clinical features with pulmonary complications. p<0.05 was deemed statistically significant. The data have been represented as n, %.

Clinical presentation	Patients without Pulmonary involvement, n=229 (%)	Patients with pulmonary involvement, n=26 (%)	Total, n=255 (%)	*P value
Headache	125 (54.6)	10 (38.5)	135 (52.9)	0.12
Nausea	100 (43.7)	10 (38.5)	110 (43.1)	0.61
Vomiting	88 (38.4)	10 (38.5)	98 (38.4)	0.99
Retro Orbital Pain	17 (7.4)	2 (7.7)	19 (7.5)	0.96
Myalgia	136 (59.4)	9 (34.6)	145 (56.9)	0.01
Arthralgia	15 (6.6)	2 (7.7)	17 (6.7)	0.82
Hemorrhage	15 (6.6)	2 (7.7)	17 (6.7)	0.82
Hemoptysis	1 (0.4)	1 (3.8)	2 (0.8)	0.06
Breathlessness	6 (2.6)	8 (30.8)	14 (5.5)	<0.01
Cough	34 (14.8)	7 (26.9)	41 (16.1)	0.11

**Figure 1:**
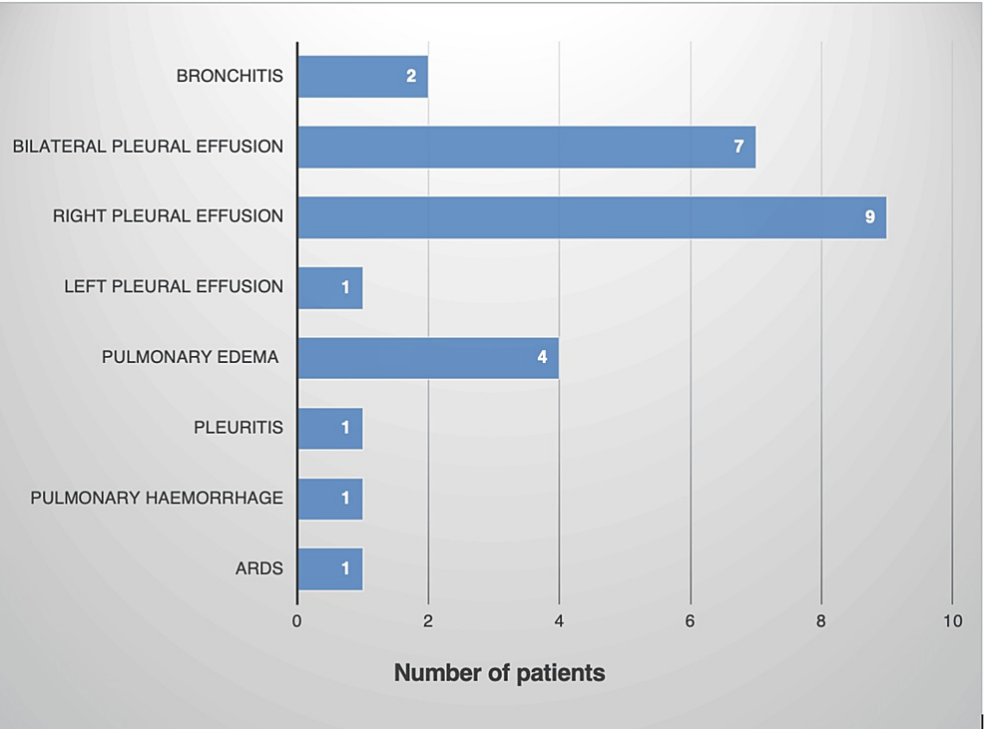
Respiratory manifestations of dengue ARDS - acute respiratory distress syndrome Data have been represented as n.

Figure 1 depicts the respiratory manifestations seen in dengue. Pulmonary complications were observed in 26 individuals, constituting 10.19% of the cases. The most prevalent manifestation was pleural effusion, present in 6.6% (n = 17) of patients, with a majority exhibiting right-sided pleural effusion (3.5%, n = 9). Additionally, upper respiratory tract involvement was noted in 5.5% (n = 14) of patients.

Table 4 presents a comparison of laboratory parameters between patients with and without pulmonary manifestations. Laboratory parameters such as total count, platelet count, urea, creatinine, total bilirubin (TB), direct bilirubin (DB), serum glutamic oxaloacetic transaminase (SGOT), and serum glutamic pyruvic transaminase (SGPT) exhibited non-normal distributions (mean < 2 standard deviations). Patients with lung involvement demonstrated higher total counts (p = 0.01), urea (p < 0.01), TB (p = 0.01), DB (p < 0.01), SGOT (p = 0.01), and SGPT (p = 0.04) levels, and lower platelet counts (p = 0.01) compared to those without pulmonary complications.

**Table 4:** Comparison of lab parameters with pulmonary complications *Mann-Whitney U test is used to compare laboratory parameters and pulmonary complications. p-value < 0.05 was deemed statistically significant. The data have been represented as n, median (Q1, Q3). ^1^Serum glutamic oxaloacetic transaminase; ^2^Serum glutamic pyruvic transaminase

Lab parameters	n	Median (Q1, Q3)	Mann-Whitney U value	*P value
Total count (cells/cumm)	254	4000 (2800, 6100)	2116.00	0.01
Platelet (cells/cumm)	255	7000 (37,000, 120,000)	2142.50	0.01
Urea (mg/dL)	255	20 (14, 26)	1206.50	<0.01
Creatinine (mg/dL)	255	1 (1, 1)	2850.50	0.42
Total Bilirubin (mg/dL)	252	1(0, 1)	2074.00	0.01
Direct Bilirubin (mg/dL)	251	0 (0, 0)	1807.50	<0.01
SGOT^1^ (U/L)	254	115 (46, 227)	1821.00	0.01
SGPT^2^ (U/L)	254	77 (32.75, 160)	2251.00	0.04

Table 5 illustrates the association between covariates and outcomes using the chi-square test of association, revealing that age over 50 years (p = 0.003), presence of comorbidities (p = 0.003), and lung involvement were associated with mortality (p < 0.05). Mortality occurred in nine patients (3.5%) with dengue.

**Table 5:** Association between covariates and outcome in the study population *Chi-square test is used to analyse the relationship between outcome and covariates such as sex, age, and comorbidities. p < 0.05 was deemed statistically significant. The data have been represented as n, %.

Covariate	Outcome		Total	*P value
	Mortality, n=9 (%)	Recovering, n=246 (%)	n=255 (%)	
Sex				
Female	1 (11.1)	77 (31.3)	78 (30.6)	0.19
Male	8 (88.9)	169 (68.7)	177 (69.4)	
Age				
≤ 50	4 (44.4)	205 (83.3)	209 (82.0)	0.003
>50	5 (55.6)	41 (16.7)	46 (18.0)	
Comorbidities				
No	4 (44.4)	204 (89.6)	208 (81.6)	0.003
Yes	5 (55.6)	42 (17.1)	47 (18.4)	
Pulmonary complications				
No	3 (33.3)	226 (91.9)	229 (89.8)	<0.01
Yes	6 (66.7)	20 (8.1)	26 (10.2)	

## Discussion

Dengue, caused by an RNA virus belonging to the Flavivirus genus, presents a diverse clinical spectrum, ranging from mild to life-threatening illness. Dengue infection encompasses a spectrum from mild dengue fever to more severe forms such as DHF and dengue shock syndrome (DSS) [12]. Clinicians often encounter diagnostic challenges when patients present with atypical symptoms. Respiratory issues are among these atypical presentations observed in clinical settings [12]. The precise prevalence of respiratory manifestation among patients with dengue infection remains uncertain. Nevertheless, several studies have indicated a higher prevalence in cases of severe dengue infection. Common respiratory manifestations include pleural effusion, pneumonitis, haemoptysis, pulmonary haemorrhage, and ARDS [8]. We conducted a study to ascertain the incidence of pulmonary manifestations among dengue patients receiving treatment at our tertiary care center situated in Mangalore, an endemic region of Karnataka, India.

In our study, we noted a reduced incidence of DHF and DSS. Out of the 255 dengue cases examined, the vast majority of patients (n = 239, 93.7%) exhibited symptoms consistent with classical dengue fever. It is worth noting that Gupta et al. reported a higher incidence of DHF (n = 22, 44%) and DSS (n = 20, 16%) in their study [13], while Mohamed et al. also presented a similar incidence of DHF (n = 42, 42%) and DSS (n = 15, 15%) [14]. Our findings indicated a higher prevalence of dengue among younger patients (82%, n = 209 vs. 18%, n = 46), consistent with previous research [14]. We observed that patients with lung manifestations tended to be older compared to those without such manifestations. Moreover, mortality rates were elevated among patients aged over 50 years.

The prevailing evidence from multiple studies suggests that individuals with conditions such as chronic chest disease, cardiac ailments, and diabetes experience higher mortality rates [13,14] and are more prone to pulmonary complications [13]. In our study group, we found that individuals with comorbidities such as hypertension, diabetes, and chronic respiratory diseases had a greater likelihood of experiencing lung involvement.

Dengue fever typically manifests with fever as the predominant symptom. In our study group, respiratory symptoms included cough, breathlessness, and haemoptysis. Notably, breathlessness was more prevalent among patients exhibiting lung manifestations. It is noteworthy that 14.8% (n = 34) of patients who did not exhibit lung manifestations reported experiencing cough. Other studies have indicated a higher frequency of cough and breathlessness in cases of dengue with lung involvement [13,14].

We observed pulmonary manifestations in 26 out of 255 patients diagnosed with dengue fever, constituting 10.19% of the cases. Pleural effusion was the most prevalent manifestation, present in 6.6% (n = 17) of patients, with a majority exhibiting right-sided pleural effusion (3.5%, n = 9). This finding was also supported by the case study conducted by Marchiori et al. [7]. Neerja et al. reported 11% (n = 19) of dengue cases with pleural effusion [15]. Gupta et al. reported a significant incidence of 56% (n = 28) of lung manifestations in their study, with ARDS being the most prevalent, exclusively observed in patients with DHF and DSS. This elevated occurrence could be attributed to a larger proportion of severe dengue cases of 60% (n = 30) in their study [13]. A comparable observation was also made by Mohamed et al., where ARDS stood out as the most common manifestation [14].

Rodrigues et al. utilized computer tomography to assess lung manifestations, finding that pleural effusion was present in 55.17% (n = 16) of patients, particularly prevalent among those with dengue fever exhibiting warning signs [9]. Within our study population, only one patient underwent a CT chest scan, which showed no abnormalities.

Comparative analysis of laboratory parameters revealed higher levels of total count, urea, TB, DB, and liver enzymes, alongside lower platelet counts in patients with lung manifestations. While studies have not extensively compared laboratory parameters between patients with and without lung involvement, common findings in dengue fever include leucopenia, thrombocytopenia, elevated liver enzymes, and creatinine [9,16].

Our study highlighted elevated mortality rates among patients with lung manifestations, comorbidities, and older age, corroborating findings from Gupta et al. [13] and Mohamed et al. [14] who also noted increased mortality among patients with comorbidities and mechanical ventilation. Moreover, Gupta et al. identified higher mortality rates among patients from low socioeconomic groups and rural settings, with risk factors including smoking, alcohol, and tobacco [13].

We encountered a few limitations in our study. Since it was retrospective, we could not incorporate subsequent clinical findings of patients during their hospitalization for the diagnosis of lung manifestations. Typically, chest X-rays and ultrasounds were conducted upon admission and were not repeated during the hospital stay unless specifically requested by the physician. While serial chest X-rays could capture dynamic lung changes, their frequent use would escalate healthcare costs. It is worth noting that our center reports a lower incidence of severe dengue compared to findings in other studies.

## Conclusions

Our study underscores the significance of identifying respiratory symptoms in dengue fever, especially in older patients and those with underlying health conditions. These manifestations frequently occur in severe forms of dengue-like DHF and DSS. Patients experiencing pulmonary symptoms have a higher mortality rate compared to those without such manifestations. Therefore, early recognition of these atypical symptoms is essential for enhancing patient prognosis.
